# 5-Pentyl-1*H*-tetra­zole

**DOI:** 10.1107/S1600536810052244

**Published:** 2010-12-18

**Authors:** Thorsten Rieth, Dieter Schollmeyer, Heiner Detert

**Affiliations:** a University Mainz, Duesbergweg 10-14, 55099 Mainz, Germany

## Abstract

The title compound C_6_H_12_N_4_, is one of a few known tetra­zoles with an alkyl chain in the 5-position. The asymmetric unit contains two independent mol­ecules. The mol­ecules are linked by N—H⋯N inter­actions into chains with graph-set notation *D*(2) and *C*_2_^2^(8) along [010]. The two independent mol­ecules form a layered structure, the layers being composed of inter­digitating strands of alternatingly oriented and nearly identical mol­ecules.

## Related literature

For synthetic methods see: Mihina & Herbst (1950 ▶); Steven *et al.* (1993 ▶); Detert & Schollmeyer (1999 ▶); Sugiono & Detert (2001 ▶); Glang *et al.* (2008 ▶); Borchmann *et al.* (2010 ▶). For the properties of tetra­zole, see: Huisgen *et al.* (1960*a* ▶,*b* ▶, 1961 ▶); Singh (1980 ▶); Pernice *et al.* (1988 ▶); Huff *et al.* (1996 ▶). For graph-set notation, see: Bernstein *et al.* (1995 ▶).
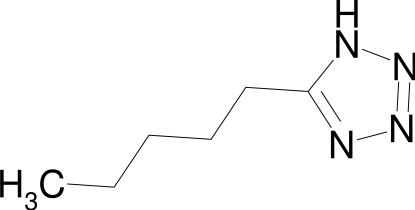


## Experimental

### 

#### Crystal data

C_6_H_12_N_4_
*M* *_r_* = 140.20
Triclinic, 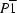

*a* = 8.7812 (14) Å
*b* = 9.6770 (12) Å
*c* = 11.614 (2) Å
α = 93.136 (10)°
β = 112.059 (9)°
γ = 116.389 (7)°
*V* = 789.6 (2) Å^3^
*Z* = 4
Cu *K*α radiation
μ = 0.63 mm^−1^
*T* = 193 K
0.50 × 0.40 × 0.30 mm

#### Data collection

Enraf–Nonius CAD-4 diffractometer
3182 measured reflections
2991 independent reflections
2764 reflections with *I* > 2σ(*I*)
*R*_int_ = 0.070
3 standard reflections every 60 min  intensity decay: 2%

#### Refinement

*R*[*F* ^2^ > 2σ(*F* ^2^)] = 0.040
*wR*(*F* ^2^) = 0.109
*S* = 1.04
2991 reflections
184 parameters
H-atom parameters constrained
Δρ_max_ = 0.24 e Å^−3^
Δρ_min_ = −0.20 e Å^−3^

### 

Data collection: *CAD-4 Software* (Enraf–Nonius, 1989 ▶); cell refinement: *CAD-4 Software*; data reduction: *CORINC* (Dräger & Gattow, 1971 ▶); program(s) used to solve structure: *SIR97* (Altomare *et al.*, 1999 ▶); program(s) used to refine structure: *SHELXL97* (Sheldrick, 2008 ▶); molecular graphics: *PLATON* (Spek, 2009 ▶); software used to prepare material for publication: *PLATON*.

## Supplementary Material

Crystal structure: contains datablocks I, global. DOI: 10.1107/S1600536810052244/bx2337sup1.cif

Structure factors: contains datablocks I. DOI: 10.1107/S1600536810052244/bx2337Isup2.hkl

Additional supplementary materials:  crystallographic information; 3D view; checkCIF report

## Figures and Tables

**Table 1:** Hydrogen-bond geometry (Å, °)

*D*—H⋯*A*	*D*—H	H⋯*A*	*D*⋯*A*	*D*—H⋯*A*
N1*A*—H1*A*⋯N4*B*	0.96	1.82	2.7773 (14)	175
N1*B*—H1*B*⋯N4*A*^i^	0.95	1.84	2.7779 (14)	170

Symmetry code: (i) 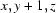.

**Fig. 1.**
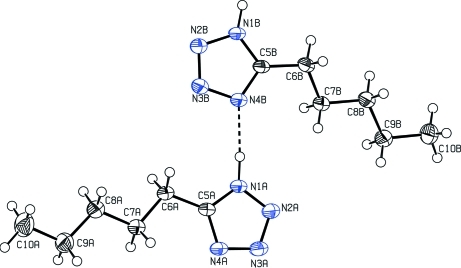
View of compound I. Displacement ellipsoids are drawn at the 50% probability level.

## supplementary crystallographic information

### Comment

The title compound (I), is formed by the addition of triethylammonium azide to capronitrile in refluxing toluene and acidic work-up. In the crystal, molecules are linked by N— H··· N interactions into chains with graph-set notation D(2) a C^2^_2_(8) along [010] (Bernstein *et al.*, 1995), Table 1. Both molecules of the title compound have very similar geometries. The heterocycles and alkyl chains are coplanar with the molecules A oriented to the opposite site of the molecules B. These strands form layers *via* interdigitation of the alkyl chains.

### Experimental

The title compound was prepared as follows: Triethyl ammonium chloride (8.95 g, 0.06 mol) and sodium azide (3.90 g, 0.06 mol) were added to a solution of hexanoic acid nitrile (4.36 g, 0.045 mol) in toluene (35 ml) and the mixture was stirred under reflux for 72 h. The mixture was filtered, the solvent evaporated and the residue dissolved in water. Hydrochloric acid (6*M*, 15 ml) was added and the product was extracted with ether/petroleum ether (1/1, 3*30 ml). The cooled organic solutions were dried with sodium sulfate. The solvents were evaporated and the residue crystallized upon standing at ambient temperature within 5 days. Recrystallization from toluene yielded 5-pentyltetrazole in 78% yield as colorless needles.

### Refinement

Hydrogen atoms attached to carbons were placed at calculated positions with C—H = 0.95 Å (aromatic) or 0.98–0.99 Å (*sp*^3^ C-atom). The Hydrogen atoms attached to N1A and N1B were located in diff. Fourier maps. All H atoms were refined in the riding-model approximation with isotropic displacement parameters (set at 1.2–1.5 times of the *U*_eq_ of the parent atom).

### Crystal data

**Table:**

C_6_H_12_N_4_	*Z* = 4
*M**_r_* = 140.20	*F*(000) = 304
Triclinic, *P*1	*D*_x_ = 1.179 Mg m^−^^3^
Hall symbol: -P 1	Melting point: 315 K
*a* = 8.7812 (14) Å	Cu *K*α radiation, λ = 1.54178 Å
*b* = 9.6770 (12) Å	Cell parameters from 25 reflections
*c* = 11.614 (2) Å	θ = 65–69°
α = 93.136 (10)°	µ = 0.63 mm^−^^1^
β = 112.059 (9)°	*T* = 193 K
γ = 116.389 (7)°	Block, colourless
*V* = 789.6 (2) Å^3^	0.50 × 0.40 × 0.30 mm

### Data collection

**Table:**

Enraf–Nonius CAD-4 diffractometer	*R*_int_ = 0.070
Radiation source: rotating anode	θ_max_ = 70.0°, θ_min_ = 4.3°
graphite	*h* = −10→9
ω/2θ scans	*k* = 0→11
3182 measured reflections	*l* = −14→14
2991 independent reflections	3 standard reflections every 60 min
2764 reflections with *I* > 2σ(*I*)	intensity decay: 2%

### Refinement

**Table:**

Refinement on *F*^2^	Secondary atom site location: difference Fourier map
Least-squares matrix: full	Hydrogen site location: inferred from neighbouring sites
*R*[*F*^2^ > 2σ(*F*^2^)] = 0.040	H-atom parameters constrained
*wR*(*F*^2^) = 0.109	*w* = 1/[σ^2^(*F*_o_^2^) + (0.0611*P*)^2^ + 0.1618*P*] where *P* = (*F*_o_^2^ + 2*F*_c_^2^)/3
*S* = 1.04	(Δ/σ)_max_ < 0.001
2991 reflections	Δρ_max_ = 0.24 e Å^−^^3^
184 parameters	Δρ_min_ = −0.20 e Å^−^^3^
0 restraints	Extinction correction: *SHELXL97* (Sheldrick, 2008), Fc^*^=kFc[1+0.001xFc^2^λ^3^/sin(2θ)]^-1/4^
Primary atom site location: structure-invariant direct methods	Extinction coefficient: 0.0088 (12)

### Special details

**Table:**

Geometry. All e.s.d.'s (except the e.s.d. in the dihedral angle between two l.s. planes) are estimated using the full covariance matrix. The cell e.s.d.'s are taken into account individually in the estimation of e.s.d.'s in distances, angles and torsion angles; correlations between e.s.d.'s in cell parameters are only used when they are defined by crystal symmetry. An approximate (isotropic) treatment of cell e.s.d.'s is used for estimating e.s.d.'s involving l.s. planes.
Refinement. Refinement of *F*^2^ against ALL reflections. The weighted *R*-factor *wR* and goodness of fit *S* are based on *F*^2^, conventional *R*-factors *R* are based on *F*, with *F* set to zero for negative *F*^2^. The threshold expression of *F*^2^ > σ(*F*^2^) is used only for calculating *R*-factors(gt) *etc*. and is not relevant to the choice of reflections for refinement. *R*-factors based on *F*^2^ are statistically about twice as large as those based on *F*, and *R*- factors based on ALL data will be even larger.

### Fractional atomic coordinates and isotropic or equivalent isotropic displacement parameters (Å^2^)

**Table:**

	*x*	*y*	*z*	*U*_iso_*/*U*_eq_	
N1A	0.76292 (14)	0.44445 (11)	0.33040 (10)	0.0321 (2)	
H1A	0.7628	0.5432	0.3397	0.038*	
N2A	0.77322 (17)	0.38035 (13)	0.22933 (10)	0.0392 (3)	
N3A	0.77338 (17)	0.25012 (13)	0.24908 (11)	0.0413 (3)	
N4A	0.76329 (16)	0.22891 (12)	0.36157 (10)	0.0358 (3)	
C5A	0.75588 (16)	0.35102 (13)	0.41070 (11)	0.0286 (3)	
C6A	0.74035 (18)	0.38071 (13)	0.53191 (11)	0.0321 (3)	
H6A	0.6195	0.3797	0.5105	0.039*	
H6B	0.8458	0.4884	0.5875	0.039*	
C7A	0.74667 (18)	0.25697 (14)	0.60683 (12)	0.0336 (3)	
H7A	0.6359	0.1501	0.5540	0.040*	
H7B	0.8633	0.2525	0.6233	0.040*	
C8A	0.74449 (18)	0.29643 (15)	0.73469 (12)	0.0360 (3)	
H8A	0.6351	0.3123	0.7186	0.043*	
H8B	0.8619	0.3983	0.7905	0.043*	
C9A	0.7308 (2)	0.16675 (18)	0.80548 (14)	0.0441 (3)	
H9A	0.6116	0.0655	0.7507	0.053*	
H9B	0.8383	0.1489	0.8197	0.053*	
C10A	0.7338 (3)	0.2091 (2)	0.93461 (16)	0.0607 (4)	
H10A	0.7183	0.1198	0.9740	0.091*	
H10B	0.6298	0.2299	0.9215	0.091*	
H10C	0.8555	0.3050	0.9916	0.091*	
N1B	0.77122 (14)	0.95284 (11)	0.40731 (9)	0.0305 (2)	
H1B	0.7639	1.0469	0.3995	0.037*	
N2B	0.74619 (15)	0.88499 (12)	0.50153 (10)	0.0350 (3)	
N3B	0.74529 (16)	0.75189 (12)	0.47932 (10)	0.0364 (3)	
N4B	0.76875 (15)	0.73261 (12)	0.37130 (10)	0.0336 (2)	
C5B	0.78464 (16)	0.85919 (13)	0.32751 (11)	0.0288 (3)	
C6B	0.81748 (19)	0.89627 (13)	0.21354 (12)	0.0359 (3)	
H6C	0.9558	0.9640	0.2423	0.043*	
H6D	0.7569	0.9590	0.1766	0.043*	
C7B	0.74053 (18)	0.74860 (13)	0.10847 (11)	0.0326 (3)	
H7C	0.8051	0.6880	0.1436	0.039*	
H7D	0.6030	0.6784	0.0813	0.039*	
C8B	0.77032 (18)	0.79245 (14)	−0.00809 (11)	0.0341 (3)	
H8C	0.7002	0.8484	−0.0453	0.041*	
H8D	0.9071	0.8678	0.0205	0.041*	
C9B	0.70495 (19)	0.64908 (15)	−0.11205 (12)	0.0380 (3)	
H9C	0.5680	0.5737	−0.1411	0.046*	
H9D	0.7749	0.5930	−0.0751	0.046*	
C10B	0.7361 (2)	0.69521 (18)	−0.22777 (13)	0.0501 (4)	
H10D	0.6736	0.5983	−0.2974	0.075*	
H10E	0.8727	0.7536	−0.2030	0.075*	
H10F	0.6822	0.7635	−0.2575	0.075*	

### Atomic displacement parameters (Å^2^)

**Table:**

	*U*^11^	*U*^22^	*U*^33^	*U*^12^	*U*^13^	*U*^23^
N1A	0.0489 (6)	0.0245 (5)	0.0357 (5)	0.0239 (4)	0.0240 (5)	0.0118 (4)
N2A	0.0627 (7)	0.0336 (6)	0.0384 (6)	0.0314 (5)	0.0295 (5)	0.0145 (4)
N3A	0.0686 (7)	0.0333 (6)	0.0397 (6)	0.0336 (5)	0.0308 (6)	0.0135 (5)
N4A	0.0564 (6)	0.0268 (5)	0.0379 (6)	0.0269 (5)	0.0264 (5)	0.0118 (4)
C5A	0.0357 (6)	0.0209 (5)	0.0337 (6)	0.0163 (5)	0.0171 (5)	0.0077 (4)
C6A	0.0446 (6)	0.0263 (6)	0.0350 (6)	0.0218 (5)	0.0219 (5)	0.0094 (5)
C7A	0.0440 (7)	0.0288 (6)	0.0360 (6)	0.0214 (5)	0.0214 (5)	0.0112 (5)
C8A	0.0426 (7)	0.0348 (6)	0.0348 (6)	0.0211 (5)	0.0193 (5)	0.0100 (5)
C9A	0.0566 (8)	0.0519 (8)	0.0428 (7)	0.0358 (7)	0.0283 (6)	0.0228 (6)
C10A	0.0868 (12)	0.0807 (12)	0.0495 (9)	0.0573 (10)	0.0426 (9)	0.0353 (8)
N1B	0.0454 (6)	0.0229 (5)	0.0326 (5)	0.0217 (4)	0.0203 (4)	0.0098 (4)
N2B	0.0511 (6)	0.0294 (5)	0.0353 (5)	0.0242 (5)	0.0242 (5)	0.0116 (4)
N3B	0.0544 (6)	0.0303 (5)	0.0370 (5)	0.0255 (5)	0.0263 (5)	0.0151 (4)
N4B	0.0520 (6)	0.0262 (5)	0.0362 (5)	0.0250 (5)	0.0256 (5)	0.0133 (4)
C5B	0.0386 (6)	0.0206 (5)	0.0312 (6)	0.0168 (5)	0.0169 (5)	0.0074 (4)
C6B	0.0554 (7)	0.0235 (6)	0.0359 (6)	0.0205 (5)	0.0260 (6)	0.0118 (5)
C7B	0.0440 (7)	0.0240 (6)	0.0350 (6)	0.0174 (5)	0.0220 (5)	0.0094 (5)
C8B	0.0457 (7)	0.0276 (6)	0.0339 (6)	0.0194 (5)	0.0206 (5)	0.0117 (5)
C9B	0.0507 (7)	0.0322 (6)	0.0347 (6)	0.0208 (6)	0.0224 (6)	0.0097 (5)
C10B	0.0734 (10)	0.0466 (8)	0.0388 (7)	0.0305 (7)	0.0324 (7)	0.0150 (6)

### Geometric parameters (Å, °)

**Table:**

N1A—C5A	1.3330 (15)	N1B—C5B	1.3339 (15)
N1A—N2A	1.3494 (14)	N1B—N2B	1.3446 (14)
N1A—H1A	0.9564	N1B—H1B	0.9474
N2A—N3A	1.2937 (14)	N2B—N3B	1.2956 (14)
N3A—N4A	1.3628 (15)	N3B—N4B	1.3616 (14)
N4A—C5A	1.3223 (14)	N4B—C5B	1.3222 (14)
C5A—C6A	1.4892 (16)	C5B—C6B	1.4868 (16)
C6A—C7A	1.5267 (16)	C6B—C7B	1.5228 (16)
C6A—H6A	0.9900	C6B—H6C	0.9900
C6A—H6B	0.9900	C6B—H6D	0.9900
C7A—C8A	1.5222 (16)	C7B—C8B	1.5203 (16)
C7A—H7A	0.9900	C7B—H7C	0.9900
C7A—H7B	0.9900	C7B—H7D	0.9900
C8A—C9A	1.5226 (18)	C8B—C9B	1.5167 (17)
C8A—H8A	0.9900	C8B—H8C	0.9900
C8A—H8B	0.9900	C8B—H8D	0.9900
C9A—C10A	1.520 (2)	C9B—C10B	1.5212 (17)
C9A—H9A	0.9900	C9B—H9C	0.9900
C9A—H9B	0.9900	C9B—H9D	0.9900
C10A—H10A	0.9800	C10B—H10D	0.9800
C10A—H10B	0.9800	C10B—H10E	0.9800
C10A—H10C	0.9800	C10B—H10F	0.9800
			
C5A—N1A—N2A	109.67 (9)	C5B—N1B—N2B	109.65 (9)
C5A—N1A—H1A	127.6	C5B—N1B—H1B	129.2
N2A—N1A—H1A	122.7	N2B—N1B—H1B	120.8
N3A—N2A—N1A	106.07 (10)	N3B—N2B—N1B	106.22 (9)
N2A—N3A—N4A	110.18 (10)	N2B—N3B—N4B	110.06 (9)
C5A—N4A—N3A	106.84 (9)	C5B—N4B—N3B	106.84 (9)
N4A—C5A—N1A	107.23 (10)	N4B—C5B—N1B	107.23 (10)
N4A—C5A—C6A	127.37 (10)	N4B—C5B—C6B	127.50 (10)
N1A—C5A—C6A	125.40 (10)	N1B—C5B—C6B	125.25 (10)
C5A—C6A—C7A	112.98 (9)	C5B—C6B—C7B	113.84 (9)
C5A—C6A—H6A	109.0	C5B—C6B—H6C	108.8
C7A—C6A—H6A	109.0	C7B—C6B—H6C	108.8
C5A—C6A—H6B	109.0	C5B—C6B—H6D	108.8
C7A—C6A—H6B	109.0	C7B—C6B—H6D	108.8
H6A—C6A—H6B	107.8	H6C—C6B—H6D	107.7
C8A—C7A—C6A	111.95 (10)	C8B—C7B—C6B	111.84 (10)
C8A—C7A—H7A	109.2	C8B—C7B—H7C	109.2
C6A—C7A—H7A	109.2	C6B—C7B—H7C	109.2
C8A—C7A—H7B	109.2	C8B—C7B—H7D	109.2
C6A—C7A—H7B	109.2	C6B—C7B—H7D	109.2
H7A—C7A—H7B	107.9	H7C—C7B—H7D	107.9
C7A—C8A—C9A	113.17 (11)	C9B—C8B—C7B	113.44 (10)
C7A—C8A—H8A	108.9	C9B—C8B—H8C	108.9
C9A—C8A—H8A	108.9	C7B—C8B—H8C	108.9
C7A—C8A—H8B	108.9	C9B—C8B—H8D	108.9
C9A—C8A—H8B	108.9	C7B—C8B—H8D	108.9
H8A—C8A—H8B	107.8	H8C—C8B—H8D	107.7
C10A—C9A—C8A	112.79 (12)	C8B—C9B—C10B	112.71 (11)
C10A—C9A—H9A	109.0	C8B—C9B—H9C	109.0
C8A—C9A—H9A	109.0	C10B—C9B—H9C	109.0
C10A—C9A—H9B	109.0	C8B—C9B—H9D	109.0
C8A—C9A—H9B	109.0	C10B—C9B—H9D	109.0
H9A—C9A—H9B	107.8	H9C—C9B—H9D	107.8
C9A—C10A—H10A	109.5	C9B—C10B—H10D	109.5
C9A—C10A—H10B	109.5	C9B—C10B—H10E	109.5
H10A—C10A—H10B	109.5	H10D—C10B—H10E	109.5
C9A—C10A—H10C	109.5	C9B—C10B—H10F	109.5
H10A—C10A—H10C	109.5	H10D—C10B—H10F	109.5
H10B—C10A—H10C	109.5	H10E—C10B—H10F	109.5
			
C5A—N1A—N2A—N3A	−0.33 (14)	C5B—N1B—N2B—N3B	0.27 (13)
N1A—N2A—N3A—N4A	0.02 (14)	N1B—N2B—N3B—N4B	−0.24 (13)
N2A—N3A—N4A—C5A	0.30 (15)	N2B—N3B—N4B—C5B	0.13 (14)
N3A—N4A—C5A—N1A	−0.49 (13)	N3B—N4B—C5B—N1B	0.04 (13)
N3A—N4A—C5A—C6A	178.87 (11)	N3B—N4B—C5B—C6B	178.27 (11)
N2A—N1A—C5A—N4A	0.52 (14)	N2B—N1B—C5B—N4B	−0.19 (13)
N2A—N1A—C5A—C6A	−178.86 (11)	N2B—N1B—C5B—C6B	−178.47 (11)
N4A—C5A—C6A—C7A	3.98 (18)	N4B—C5B—C6B—C7B	28.32 (18)
N1A—C5A—C6A—C7A	−176.77 (11)	N1B—C5B—C6B—C7B	−153.76 (12)
C5A—C6A—C7A—C8A	176.01 (10)	C5B—C6B—C7B—C8B	177.66 (10)
C6A—C7A—C8A—C9A	174.26 (11)	C6B—C7B—C8B—C9B	176.94 (11)
C7A—C8A—C9A—C10A	178.51 (12)	C7B—C8B—C9B—C10B	−179.91 (11)

### Hydrogen-bond geometry (Å, °)

**Table:**

*D*—H···*A*	*D*—H	H···*A*	*D*···*A*	*D*—H···*A*
N1A—H1A···N4B	0.96	1.82	2.7773 (14)	175
N1B—H1B···N4A^i^	0.95	1.84	2.7779 (14)	170

Symmetry codes: (i) *x*, *y*+1, *z*.
